# Evaluation of a MetOp ASCAT‐Derived Surface Soil Moisture Product in Tundra Environments

**DOI:** 10.1029/2018JF004658

**Published:** 2018-12-06

**Authors:** Elin Högström, Birgit Heim, Annett Bartsch, Helena Bergstedt, Georg Pointner

**Affiliations:** ^1^ Austrian Polar Research Institute Vienna Austria; ^2^ Vienna University of Technology Vienna Austria; ^3^ Alfred Wegener Institute for Polar and Marine Research Potsdam Germany; ^4^ b.geos Korneuburg Austria; ^5^ Department of Geoinformatics‐Z_GIS University of Salzburg Salzburg Austria

**Keywords:** radar, tundra, soil moisture, C band, Arctic

## Abstract

Satellite‐derived surface soil moisture data are available for the Arctic, but detailed validation is still lacking. Previous studies have shown low correlations between in situ and modeled data. It is hypothesized that soil temperature variations after soil thaw impact MetOp ASCAT satellite‐derived surface soil moisture (SSM) measurements in wet tundra environments, as C band backscatter is sensitive to changes in dielectric properties. We compare in situ measurements of water content within the active layer at four sites across the Arctic in Alaska (Barrow, Sagwon, Toolik) and Siberia (Tiksi), taken in the spring after thawing and in autumn prior to freezing. In addition to the long‐term measurement fields, where sensors are installed deeper in the ground, we designed a monitoring setup for measuring moisture very close to the surface in the Lena River Delta, Siberia. The volumetric water content (VWC) and soil temperature sensors were placed in the moss organic layer in order to account for the limited penetration depth of the radar signal. ASCAT SSM variations are generally very small, in line with the low variability of in situ VWC. Short‐term changes after complete thawing of the upper organic layer, however, seem to be mostly influenced by soil temperature. Correlations between SSM and in situ VWC are generally very low, or even negative. Mean standard deviation matching results in a comparably high root‐mean‐square error (on average 11%) for predictions of VWC. Further investigations and measurement networks are needed to clarify factors causing temporal variation of C band backscatter in tundra regions.

## Key Points

- Organic layer temperature variations explain temporal behavior of soil moisture derived from C band radar in unfrozen wet tundra
- Temporal variability of volumetric water content from in situ point measurements is representative over distances of several kilometers in tundra
- Near‐surface volumetric water content predicted from matched ASCAT data has an average RMSE of 11% across five sites in Alaska and Siberia

## 1 Introduction

The Arctic landscape is characterized by its heterogeneity (e.g., Bartsch et al., 2016; Fletcher et al., 2010), its many surface water bodies (Smith et al., 2007), the presence of permafrost in the ground and frozen surface conditions for the majority of the year (Zhang et al., 2003). The soil layer close to the surface, just above the permafrost table, which experiences seasonal freezing and thawing is referred to as the active layer. In summer, the thaw front penetrates from the surface downward and the active layer deepens as positive soil temperatures prevail. In autumn, the freeze front penetrates downward from the surface, as well as upward from the permafrost table. Knowledge about the amount of water that freezes in the active layer in autumn is of interest, as this water will be available as potentially liquid water for the following spring. This information has been shown to be valuable, for example, for wildfire prediction and is of interest for purposes related to heat transfer from and isolation of the frozen ground (Beer et al., 2007). Ice has a lower thermal conductivity than water. Therefore, the active layer is expected to isolate the underlying permafrost better if the water fraction in the soil is high at the time of freezing in autumn. Soil moisture data are generally in high demand in this region; for example, for permafrost‐related applications, specifically modeling and flux upscaling studies (Jung et al., 2010; Marchenko et al., 2008; Zhang et al., 2011).

A wide range of global satellite‐derived soil moisture products are available today from microwave sensors, such as the Advanced Microwave Scanning Radiometer for EOS (AMSR‐E) (Njoku et al., 2003; Owe et al., 2008), Tropical Rainfall Measuring Mission (TRMM) Microwave Imager (TMI; Owe et al., 2008), Special Sensor Microwave/Imager (SSM/I; Owe et al., 2008), WindSat Polarimetric Radiometer (Li et al., 2010), European Remote Sensing Satellites ERS 1 and 2 (Scipal et al., 2002; Wagner, Lemoine, et al., 1999), and the Advanced Scatterometer (ASCAT) onboard the Meteorological Operational satellite (MetOp; Bartalis et al., 2007; Wagner et al., 2010). More recent data collection programs include the Soil Moisture and Ocean Salinity Mission (SMOS) of the European Space Agency (Kerr et al., 2001) and the Soil Moisture Active and Passive of the United States National Aeronautics and Space Administration (NASA; Entekhabi et al., 2010), which were specifically designed for the purpose of soil moisture retrieval. Global maps of near surface soil moisture are produced using coarse resolution (∼25–50 km) sensors operating in the microwave frequencies, employing passive as well as active systems.

C band scatterometer information is of specific interest in heterogeneous environments due to the availability of higher spatial resolution Synthetic Aperture Radar (SAR) data at this wavelength. SAR can be used to assess the spatial variability and representativity of the scatterometer information (Wagner et al., 2008). The C band scatterometer ASCAT provides operational data in near real time since 2008 (Bartalis et al., 2007; Wagner et al., 2010). The microwave backscatter variations are expected to correspond to soil moisture variations. Surface roughness and volume scattering, which also contribute to the backscatter signal, are parameterized or assumed to be constant under certain conditions (Bartalis et al., 2007; Naeimi, Bartalis, et al., 2009; Naeimi, Scipal, et al., 2009). The measurements available during frozen and snow cover conditions are masked out because soil moisture data, as absolute values or saturation, can only be obtained during the unfrozen period. In addition, the existence of surface water within the scatterometer footprint poses a challenge (Wagner et al., 2013). The water surface roughness can quickly increase at high wind speeds and thus introduce a bias in lake‐rich areas (Högström & Bartsch, 2017).

A multitude of studies have validated soil moisture detection approaches through comparison with in situ measurements (Rudiger et al., 2007). The challenge is to compare the point observations of measurement stations to large scatterometer footprints of 25‐ to 50‐km size (Wagner et al., 2013).

In situ measurements are essential for evaluating satellite‐based soil moisture estimates and to understand what is represented in the backscatter signal (Ceballos et al., 2005; Wagner et al., 2007). Soil moisture measurements have been used for satellite validation purposes since the 1980s (Wang et al., 1980), and the availability of remotely sensed soil moisture from, for example, SMOS, AMSR‐E, and WindSat have led to further validation activities such as the installation of new in situ soil moisture networks (Delwart et al., 2008; Wagner et al., 2007). There is increasing availability of in situ soil moisture data (Krauss et al., 2010), and there have been attempts to collect and disseminate soil moisture data from several networks (Robock et al., 2000). In 2011, the International Soil Moisture Network (ISMN) was established, which is a centralized hosting facility of in situ soil moisture data (Dorigo et al., 2013). Soil moisture observation networks have been installed and used for calibration/validation activities for the Soil Moisture Active Passive Mission (Colliander et al., 2017), the SMOS (Bircher et al., 2012) missions, as well as the European Space Agency Climate Change Initiative soil moisture product (Ikonen et al., 2016). The above mentioned efforts are highly valuable for quantitative assessments of global remote sensing products, but the networks themselves were not always installed for that purpose, which can mean that multiple measurements within a satellite footprint are not provided (Crow et al., 2012). The large spatial variability in soil moisture (Western et al., 2002) is still challenging, and there is a need to understand the variable satellite‐derived soil moisture product quality across different environments (De Jeu et al., 2008; Dirmeyer et al., 2004; Sinclair & Pegram, 2010). Small‐scale networks also exist with relatively high spatial density (e.g., 3–5 km^2^; Jackson et al., 2010). In situ measurements overall are still concentrated in the middle and low latitudes, and there are clearly gaps to be filled across the Arctic.

Scaling between local in situ measurements and 25‐ to 50‐km footprints for scatterometer and microwave radiometer measurements remains problematic for validating results, in particular, due to the spatial and temporal variability of soil moisture (Western et al., 2002). It has been shown that the spatial variability increases with the size of the area up to 10 km^2^ but remains relatively constant thereafter and that a small number of measurements are sufficient to obtain accurate areal measurements (Brocca et al., 2012). The difference in depth between the in situ sensor, commonly installed at 10 cm or deeper, and the top layer through which the microwave signal can penetrate is crucial. The microwave signal penetration depth also depends on the moisture level itself (deeper penetration for low percentages of water). In fact, correlations between satellite and in situ measurements are usually not very high. Bartsch, Trofaier, et al. (2012) demonstrated that Pearson correlations for sensors deeper than 10 cm are below 0.4 in tundra environments. But even a lack of correlation does not necessarily mean that the satellite data are wrong. It may, for example, be explained by the fact that the point measured in situ data are not representative for a larger area, such as that of the satellite footprint of the remote sensing data product. The results need to be interpreted in their relative context. Direct comparisons between in situ data and remote sensing products remain an important aspect of validating spatial scaling of predictions and understanding small‐scale moisture variations in specific land types. When interpreting the results of a direct comparison, it is also important to consider the accuracy and quality of the in situ data set (Wagner et al., 2013).

Previous studies that compared in situ water content with satellite data indicate that the shallow installation of sensors is an appropriate choice (Bartsch et al., 2011, 2012). The uppermost sensors are more directly affected by the atmospheric conditions than those in deeper soil layers, where changes in moisture and temperature are slower and damped. Prior estimations of in situ unfrozen water content in tundra over time have been conducted using bulk electrical conductivity and in situ soil temperature data in Alaska, demonstrating the potential to remotely *visualize* permafrost via autonomous monitoring over field‐relevant scales (Dafflon et al., 2017).

In this study, in situ data records from four existing monitoring sites in northern Alaska and in Siberia, Russia, were used to investigate how in situ soil moisture data compare with the ASCAT SSM during the period of freezing in autumn and thawing in spring. Additionally, a setup of in situ temperature and soil moisture sensors has been developed specifically for the purpose of ASCAT assessment. Sensors have been installed in a high‐latitude Arctic location in order to investigate the validity of near‐surface soil moisture retrieved with the ASCAT instrument. It is hypothesized that although absolute values may differ between the in situ measurement sites within the ASCAT footprint, the variation in measured values will be similar. The integrated measurement from a certain ASCAT footprint area is expected to reflect the general moisture level in these landscapes. It is further hypothesized that soil temperature variations during the transition period are reflected in MetOp ASCAT measurements as the dielectric constant of the water within the soil changes with the changing soil temperature (Hallikainen et al., 1985) and in turn influences the microwave backscatter (Naeimi et al., 2012; Ulaby & Long, 2014). Lastly, we investigate whether the ASCAT SSM measurements before freeze‐up are valid and to what extent the ASCAT SSM is related to the soil temperature development.

## 2 Materials and Methods

### 2.1 MetOp ASCAT Soil Moisture Product

ASCAT is a fan‐beam scatterometer with six side‐looking antennas that illuminate two 550‐km‐wide swaths on each side of the satellite track, separated by a gap of 360 km. The instrument is onboard the series of polar orbiting Meteorological Operational (MetOp) satellites: MetOp‐A, launched in 2006, MetOp‐B in 2012, and MetOp‐C is planned to be launched in 2018. The MetOp constellation flies in a near‐polar Sun‐synchronous orbit with a repeat cycle of 29 days and completing 14 orbits per day. The daily global coverage with one MetOp is about 82%, with full coverage over the poles (>65°), where three to four acquisitions are available per day by combining the ascending and descending orbits, for example, at approximately 2, 5, 9, and 12 p.m. UTC in the Lena Delta, Siberia.

When ASCAT was designed its main purpose was to measure wind speed and direction over the oceans (Figa‐Saldaña et al., 2002), but quite soon it turned out that unforeseen land applications were possible as well. EUMETSAT (European Organisation for the Exploitation of Meteorological Satellites) in cooperation with the Vienna University of Technology (TU Wien) have developed a soil moisture retrieval algorithm which is implemented in a software package called the Water Retrieval Package (WARP). Since 2008 near‐real‐time (within ∼135 min after actual acquisition) measurements of SSM are available globally from ASCAT (Bartalis et al., 2007; Wagner et al., 2010). The instrument operates at a frequency of 5.3 GHz (C band), both transmitting and receiving electromagnetic waves in vertical polarization (VV). The ASCAT incidence angle ranges from 25° to 65°, and the backscatter is normalized to a standard incidence angle of 40° (Bartalis et al., 2007). A contrast in dielectric properties between dry and wet soils leads to a strong dependency of C band backscatter intensity on the moisture content in the uppermost soil layer. This contrast forms the basis of the retrieval method. The penetration depth for ASCAT measurements is approximately 5 cm but is known to be higher for dry compared to wet soils. The reason is that water is not only a strong scatterer but also a strong absorber of low‐frequency microwaves (Schanda, 1986). The penetration depth increases almost exponentially in water depleted soil (Williams & Greeley, 2001). Besides moisture, the radar signal is also affected by surface roughness, vegetation as well as ice, and snow (Bartsch, 2010; Bartsch et al., 2011; Naeimi et al., 2012). For the ASCAT surface soil moisture retrieval a change detection approach has been developed which assumes roughness and land cover to be stable in time at a given spatial scale (Wagner, Noll, et al., 1999; EUMETSAT repository). Since scattering from vegetation is enhanced at large incidence angles and during the vegetation season, it is corrected for by using the multiincidence angle‐viewing capacities of ASCAT. The backscatter values are scaled between a dry (0%) and wet (100%) reference, corresponding to the locally obtained historical minimum and maximum values, resulting in a relative measurement of the water content (saturation) in the uppermost soil layer (Wagner, Noll, et al., 1999). Validation results for ASCAT have been overall positive, but there are regions that are problematic, such as mountains, deserts, or those with a high water fraction (Högström & Bartsch, 2017; Wagner et al., 2013).

The ASCAT time series data are available for a predefined discrete global grid which is based on the assumption that the Earth can be modeled as a rotated ellipsoid. The grid cells are spatially resampled with an equal spacing of 12.5 km in latitude and longitude and identified by a unique grid cell index (GPI). Each grid cell over land is attributed a time series of backscatter values as well as SSM data (Naeimi, Scipal, et al., 2009). Time series have been extracted from the EUMETSAT Data Center for each location for which in situ data have been available.

Frozen conditions are indicated through a so‐called Advisory Flag that ranges from 0 to 100 (unfrozen to frozen), which is available for the SSM data user. An Advisory Flag also exists for snow probability, ranging from 0 to 3 (snow‐free to snowy conditions). The flags are both based on analyses of historical data (SSM/I snow cover data and ERA‐40 climate data, respectively) and give the probability for snow and/or frozen ground for each day of the year (Nolin et al., 1998; Uppala et al., 2005). This probability does not vary from year to year. However, timing of freezing and thawing can be highly variable (Naeimi et al., 2012). For the present study all frozen and snow‐covered conditions are therefore masked out based on the in situ records.

In order to detect thaw and freeze onset dates, a 7‐day median‐filter is applied on the in situ time series of volumetric soil moisture. The thaw and freeze onsets are defined by the first and last days of the year, respectively, where the filtered time series exceed 20%. All ASCAT measurements before thaw and after freeze onsets were masked out.

### 2.2 In Situ Soil Temperature and Volumetric Water Content Records

**Figure 1:**
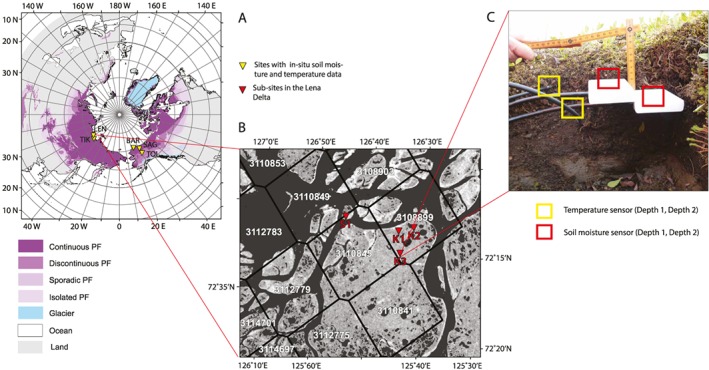
(a) In situ site locations in the Arctic overlain with a simplification (not including subclasses for ground ice content) of the Circum Arctic map of permafrost and ground ice conditions as background (Brown et al., 2002). Projection: Lambert Azimuthal. Classes contained are continuous, discontinuous, sporadic, and isolated permafrost as well as glacier, ocean, and land. (b) ENVISAT ASAR Wide Swath image for the central Lena River Delta, Siberia. The location of the installed stations on the Kurungnakh island in the Lena Delta is indicated in red. Black hexagons represent the approximate ASCAT footprint and are labeled with the ASCAT product ID. Projection: UPS North Stereographic. (c) Setup of the VWC and soil temperature sensor installation at the sites on Kurungnakh: VWC and soil temperature sensors at Depth 1 (SSM D1, T D1) (a depth of 3 cm) and at Depth 2 (SSM D2, T D2) (a depth of 6–7 cm). ENVISAT = Environmental Satellite; ASAR = Advanced Synthetic Aperture Radar; WS = Wide Swath; ASCAT = Advanced Scatterometer; UPS = universal polar stereographic; VWC = volumetric water content; SSM = surface soil moisture.

Four sites where in situ soil moisture was already available were selected in Alaska and in Siberia, Russia, for comparison between ASCAT derived SSM and in situ moisture data. All four sites, Barrow, Sagwon and Toolik in Alaska, as well as Tiksi in Siberia are long‐term permafrost monitoring sites. The data for Alaska were available through the Natural Resources Conservation Service and for Tiksi through the Finnish Meteorological Institute in Helsinki, Finland. Given that the C band sensors' penetration depth is less than 5 cm the records from in situ sensors at shallow depths correspond well to the ASCAT data records compared to those from deeper installed sensors (Bartsch, Melzer, et al., 2012). Therefore, although data for more than one depth were available from the Alaskan sites and Tiksi, only records from sensors at shallow depths were included. Figure 1 and Table 1 give an overview of the sites location, the included sensor depth, and the in situ time period covered at the specific sites which also correspond to that of ASCAT measurements (from 2007). For the selected sites, sufficiently continuous soil temperature measurements are only publicly available for Barrow on the Global Terrestrial Network for Permafrost Database (GTN‐P). The record ends in 2008, providing two years of overlap with ASCAT measurements.

**Table 1:** Table Overview of the Five Arctic Sites: Their Location, the Sensor Depth, and Brief Description of the Site

		Sensor	Data	
Sites	Location	depth (cm)	availability	Description
Toolik	68°37'22.9''N	9	2007–2011	Tussock tundra, inland,
	149°36'35.4''W		daily	south of Toolik lake.
Sagwon	69°24'08.9''N	10	2007–2011	Moist acidic tundra,
	148°47'52.9''W		daily	upland, inland.
Barrow	71°18'27.7''N	10	2007–2011	Tussock tundra, Coastal
	156°35'19.7''W		daily	lowland, polygon landscape.
Tiksi	71°35'39.48''N	5	2011–2014	Mid‐wet tundra, Coast,
	128°53'17.4''E		hourly	hilly with exposed bedrock.
Lena Delta	(see below)		2013–2014	Tundra, high water fraction
			daily
Lena Delta	Location	Sensor	Profile	Average VWC
Sub sites^a^		depth (cm)^b^		before freeze‐up 2013
K1	72°19'43.88''N	D1:3	moss layer	0.04
	126°14'59.10''E	D2: 6	fibric layer	0.45
K2	72°18'25.11''N	D1:3	moss layer	0.07
	126°12'53.50''E	D2: 8	fibric layer	0.32
K3	72°21'14.23''N	D1:3	moss layer	0.31
	126°08'26.52''E	D2: 6	fibric layer	0.54
S1	72°22'59.71''N	D1:4	moss layer	0.04
	126°28'10.39''E	D2:10	fibric layer	no data

*Note*. VWC = volumetric water content.

a K1‐3 and S1 stand for Kurungnakh one to three and Samoylov 1.

b D1 and D2 stand for depth one and two.

To get additional data on soil temperature and VWC automatic stations measuring soil temperature and VWC were deployed in the central Lena River Delta, Siberia. They were installed at a very shallow depth and intended for comparison with ASCAT measurements on the islands Kurungnakh and Samoylov (Figure 1). The Lena Delta is underlain by continuous permafrost between about 400 and 600 m below the surface (Yershov et al., 1991). It offers a large suite of different permafrost landscape types. At 72°N the landscape north of the treeline is characterized by a shrubby tundra on top of the Yedoma Islands and by polygonal wet tundra on the Holocene river terraces of the Lena Delta. Samoylov is a small island located within one of the main Lena River channels, where long‐term monitoring, including climate and soil measurement stations, has been in progress since 1996 (Boike et al., 2013). The Samoylov research station with its facilities, as well as the long study records, makes this a key site for polar research in the Siberian Arctic. Samoylov Island is dominated by a polygonal wet tundra landscape. The Yedoma landscape unit Kurungnakh is located only a few kilometers south of Samoylov Island. The measurement stations were installed in August 2013 on Kurungnakh and Samoylov and data were collected in August 2014. Three stations were placed on Kurungnakh (K1, K2, and K3) and one on Samoylov (S1). Each station on Kurungnakh consisted of (a) one VWC Campbell Recording Sensor CR625 and one Temperature T109 sensor at a shallow depth, referred to as depth one (W1 and T1), (b) one VWC CR625 sensor and one T109 sensor at a deeper depth, referred to as depth two (W2 and T2; Figure 1c). The station on Samoylov had the same setup as that on Kurungnakh, with the exception that only one depth could be instrumented (W1 and T1). The sensors at depth one were placed in the lower end of the uppermost porous moss layer (at an average of 5‐ to 7‐cm thickness). The sensors at depth two were placed in the moss fibric layer, a thin layer of ∼2 to 3 cm, which is the water storage layer of the moss and always water saturated (Yoshikawa et al., 2004). Below the fibric layer very decomposed old moss and roots from the vascular plant cover frequently form a fibric peat layer of approximately 8 to 10 cm transitioning into mineral‐dominated soil. The fibric layer may also directly overlie mineral soil of the active layer. The precise locations of each sensor are given in Table 1. Soil temperature and VWC were recorded at daily intervals. Figure 1 shows the site overview of field work in 2013 in the Lena Delta (a) and a photo of the installation (c).

### 2.3 Comparison of in Situ and Satellite Records

The temporal sampling of in situ data across the Arctic varied between measurement sites, with daily sampling in Toolik, Sagwon, Barrow, and the Lena Delta. Hourly sampling was available only for the Tiksi data. For the comparison between ASCAT SSM and in situ VWC data, daily means were used for each site and grid point.

In order to compare the ASCAT SSM (percent saturation) and in situ VWC data, two different scaling methods were employed. In the first method, the in situ data are adjusted to match ASCAT SSM values. ASCAT SSM values are determined by scaling each backscatter value to the historical maximum and minimum at that point. The in situ VWC data were therefore likewise scaled to the measured minimum and maximum in situ data. It should be noted that in the Lena Delta, the historical minimum and maximum could only be calculated from 1 year, which were then compared with a 7‐year record of ASCAT data combined with historical ERS data (Naeimi, Scipal, et al., 2009). The still mostly frozen soil and permafrost beneath impede percolation of water into the ground. Therefore, it is generally expected that soils are fully saturated after the spring snow melt in tundra environments. The fact that only the near surface is observed here needs to be considered, since this may consist of a green moss layer and thus dry up quickly. In the second comparison method, the ASCAT data are rescaled to the in situ observations using mean standard deviation matching (Brocca et al., 2011), implemented in the Python Toolbox for the Evaluation of Soil Moisture Observations (pytesmo, Paulik et al., 2014). Matching allows determination of the root‐mean‐square error (RMSE) for predicting VWC from ASCAT SSM. The Pearson correlation has additionally been derived for all sites. All statistical analyses are carried out separately for the beginning and the end of the unfrozen season, which has been defined by a 20‐day period after the thaw onset and prior to the freeze onset detected from the in situ data. The distribution of the in situ and ASCAT records (for both scaling methods, in situ scaled on own minimum‐maximum and ASCAT rescaled to in situ using mean standard deviation matching) are further assessed with boxplots for the Lena Delta and Barrow over these time periods.

The representativeness of the in situ data for the four stations in the Lena Delta was investigated by comparing the records from each station with each other. The Pearson correlation coefficient was calculated between the records from each sensor (Table 2). In situ data from the Lena Delta were collected from 2 September 2013 to 15 August 2014. The ASCAT SSM from this time period is applicable only during unfrozen and snow‐free conditions. The thaw onset was here defined using data from K1W2, as it showed the steepest increase of VWC of all the sensors. The same median‐filter and threshold of 20% as described above in section 2.1. was used. The last day used in the analysis is defined by the in situ data availability (15 August 2014). Thus, the last 20‐day period for the Lena Delta is termed *late summer*.

**Table 2:** Pearson Correlation Matrix for Intercomparisons of (top) in Situ Soil Temperatures From the Different Stations, (middle) in Situ Volumetric Water Content (VWC) From the Different Stations, and (bottom) ASCAT‐Derived Surface Soil Moisture (SSM) From Four Neighboring GPI

In situ sensor VWC						
	K2 W1	K3 W1	S1 W1	K1 W2	K2 W2	K3 W2
K1 W1	0.980	0.902	0.944	0.937	0.890	0.811
K2 W1		0.902	0.935	0.914	0.838	0.759
K3 W1			0.842	0.890	0.785	0.786
S1 W1				0.819	0.740	0.686
K1 W2					0.935	0.913
K2 W2						0.932
In situ sensor soil temperature						
	K2 T1	K3 T1	S1 T1	K1 T2	K2 T2	K3 T2
K1 T1	0.995	0.990	0.993	0.998	0.988	0.978
K2 T1		0.989	0.997	0.997	0.997	0.987
K3 T1			0.989	0.988	0.981	0.985
S1 T1				0.995	0.995	0.988
K1 T2					0.994	0.987
K2 T2						0.991
ASCAT SSM						
	3110841	3110845	3110849			
3108899	0.95	0.96	0.95			
3110841		0.95	0.91			
3110845			0.95			

Dependencies between soil temperature and the observed ASCAT backscatter over time have been analyzed for the stations on Kurungnakh and Barrow, based on Pearson correlations over a 20‐day period. This duration allows for sufficient samples and at the same time can provide information on changes of this relationship over time, considering the short unfrozen period at the Arctic sites. The Pearson correlation has been derived for the original data as well as detrended versions in order to investigate short‐term variability. Sensor soil temperature at depth one and two, as well as satellite records, have been temporally averaged over a 3‐day window for this purpose. Three‐day windows are often applied to satellite‐derived soil moisture records to reduce noise (e.g., Massari et al., 2017). This noise may, however, result from soil temperature variations. The resulting time series have been subtracted from the original data in order to exclude the long‐term variations. The correlation analyses for the 20‐day period has been repeated with the detrended data set. The same procedure has been applied to the records from Barrow.

## 3 Results

### 3.1 Soil Moisture and Temperature Dynamics in the Lena Delta

Results from the Pearson correlation analyses showed that for the temporal dynamics of the measured soil temperature in the moss and fibric peat layer the overall agreement between the sensors of the four stations is high (0.978–0.998). Table 2 shows the Pearson correlation matrix between the measured soil temperature at in situ sites. Station K3 with K3T1 and T2 showed the lowest agreement with other sensors (0.978–0.991) and sensors at similar depths showed slightly higher correlations than between different depths. The Pearson correlation for the VWC measurements range from medium to high (0.686–0.980). The lowest values were again from the K3 station (0.686–0.932). For the VWC measurements, the sensors at similar depths clearly showed higher correlations than sensor comparisons between different layers. The VWC record of the uppermost measurement in the moss layer that is in direct exchange with the atmosphere shows the highest temporal dynamics. The moss layer VWC measurements were lower than the VWC in the water‐saturated fibric layer, whereas the soil temperature was higher in the moss layer. The VWC record of the fibric layer showed a reduced expression of the same temporal dynamics with much higher wetness. The intercomparisons of SSM from the different ASCAT GPI showed overall high agreement (0.86–0.97). The GPI 3110845 showed the highest agreement with 3108899 and 3110841 (0.97).

**Figure 2:**
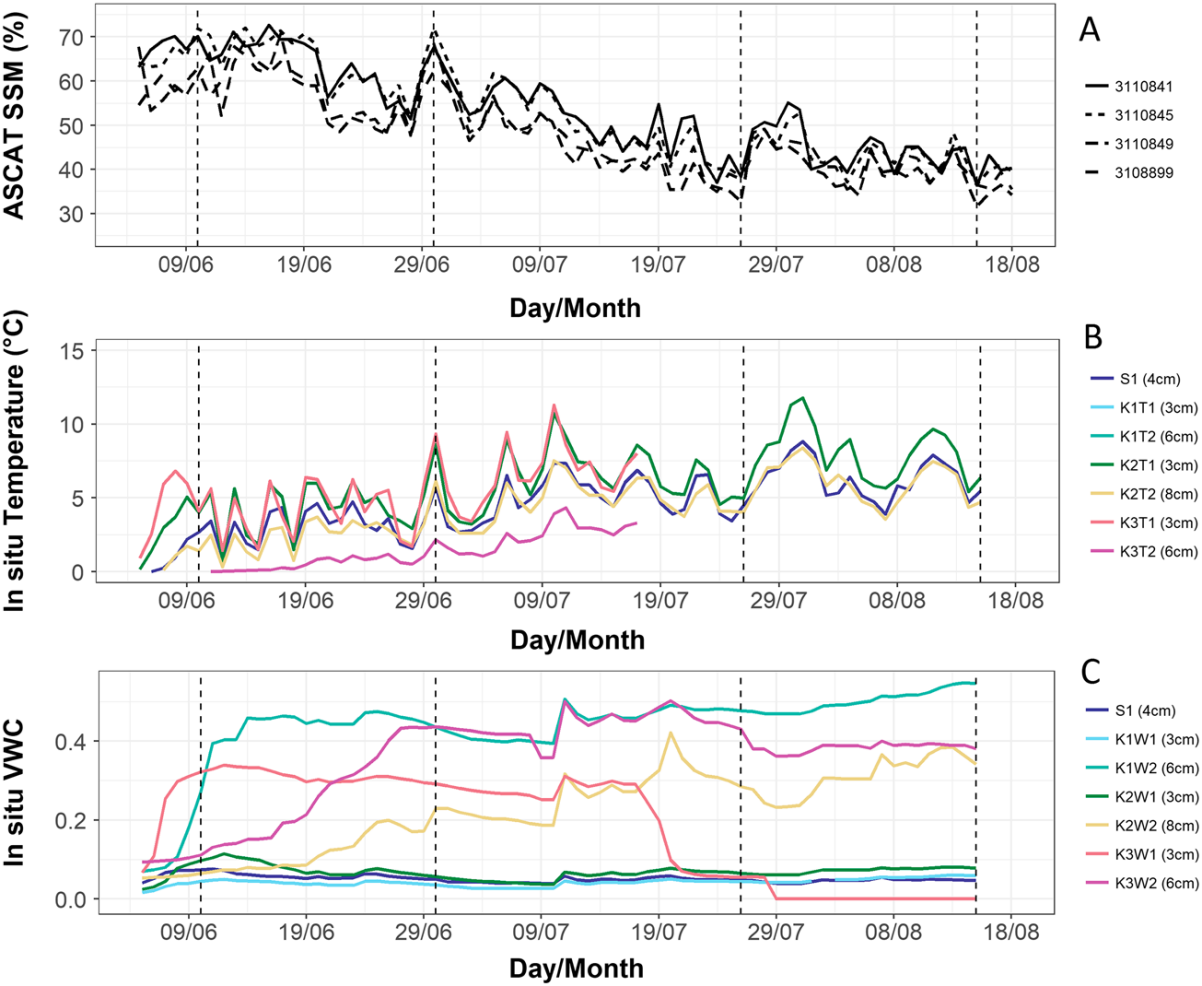
Time series of (a) Advanced Scatterometer (ASCAT) surface soil moisture (SSM), (b) in situ soil temperature, and (c) in situ volumetric water content (VWC) measured in the organic layer (moss and fibric layers) at the Lena River Delta during summer 2014. The curves in panel (a) are for different ASCAT grid points. The soil temperature shown in panel (b) is recorded at each station (K1–K3, S1) at the depth 3 to 4 cm (T1, moss layer) and 6–8 cm (T2, fibric layer). The VWC shown in panel (c) is recorded at each station (K1–K3, S1) at the depth 3 to 4 cm (T1, moss layer), and 6–8 cm (T2, fibric layer). In situ depths and ASCAT GPI are shown according to the legend. Vertical dashed lines indicate the beginning and end of time periods used for analyses in Figures 5 and 6 and Table 3.

**Figure 3:**
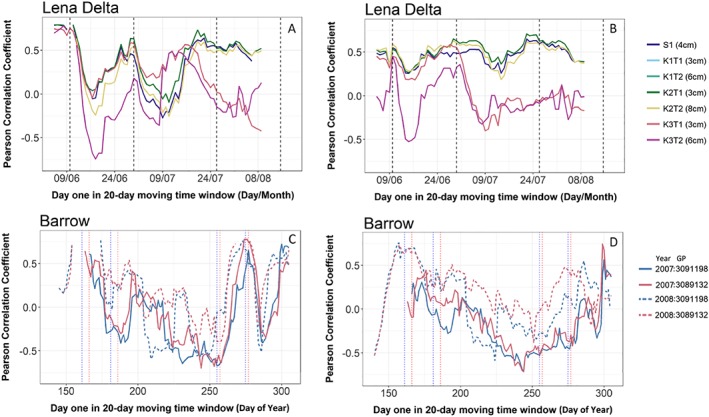
Temporal development of the Pearson correlation between ASCAT SSM and in situ soil temperature time series. (a, b) Lena Delta (GPI 3110845), (c, d) Barrow (3091198 over Barrow with ocean in ASCAT footprint, 3089132 south of Barrow). (left) Results of a 20‐day moving time window applied for the full unfrozen summer period. (right) Temporal development of the correlation between detrended ASCAT SSM and in situ soil temperature time series—results of a 20‐day moving time window applied for the full unfrozen summer period. Vertical dashed lines indicate the beginning and end of time periods used for analyses in Figures 5 and 6 and Table 3. ASCAT = Advanced Scatterometer; SSM = surface soil moisture.

The daily recorded in situ soil temperature data showed similar variations as the closest in time daily recorded ASCAT surface moisture (Figure 2). The soil temperature variations for the deeper sensor K3 as well as the soil temperature itself were much lower than for the other sites in the Lena Delta. The Pearson correlation for the 20‐day time windows ranged from approximately −0.7 to 0 in early spring, followed by a peak of 0.6 at the end of June (sensor K2 and K3, Figure 3). Correlations were also positive and medium high (values up to 0.6) in summer (mid July) but in some cases (K3) gradually became lower toward the time of freeze‐up in autumn. For the detrended data set, correlations with organic layer soil temperature were comparably high during the first 2 weeks, except for K3 at 6‐cm depth. They ranged from 0.4 to 0.5 (Figure 3). Correlations decreased for the 20‐day periods starting after approximately 10 July 2014 in the case of K1 and K2. This period is characterized by moisture variations (in situ) as shown in Figure 2. The period of higher correlation ends at the end of June for K3. Figure 2 also shows the ASCAT SSM for additional GPI locations near the sites with in situ observations. The variations of in situ VWC in the case of all the Lena Delta sites were not well represented by ASCAT SSM during the time period of in situ data availability. A period of lower moisture could be observed toward the end of July at most in situ locations, which coincides with higher SSM/backscatter. Station K1 behaved differently than the other sites in August, displaying increasing VWC. However, a late August expedition to this site showed local changes due to water logging. The installation of the sensors in 2013 seems to have triggered the degradation of this site 1 year later. The VWC sensor of station K2 ceased to operate in mid‐July 2014 due to disturbance by animals. The soil temperature records for K3 are shorter than for other sites due to damage to the sensors by rodents. Also, the moisture sensor measurements are disturbed afterward.

### 3.2 Soil Moisture and Temperature Dynamics in Alaska

**Figure 4:**
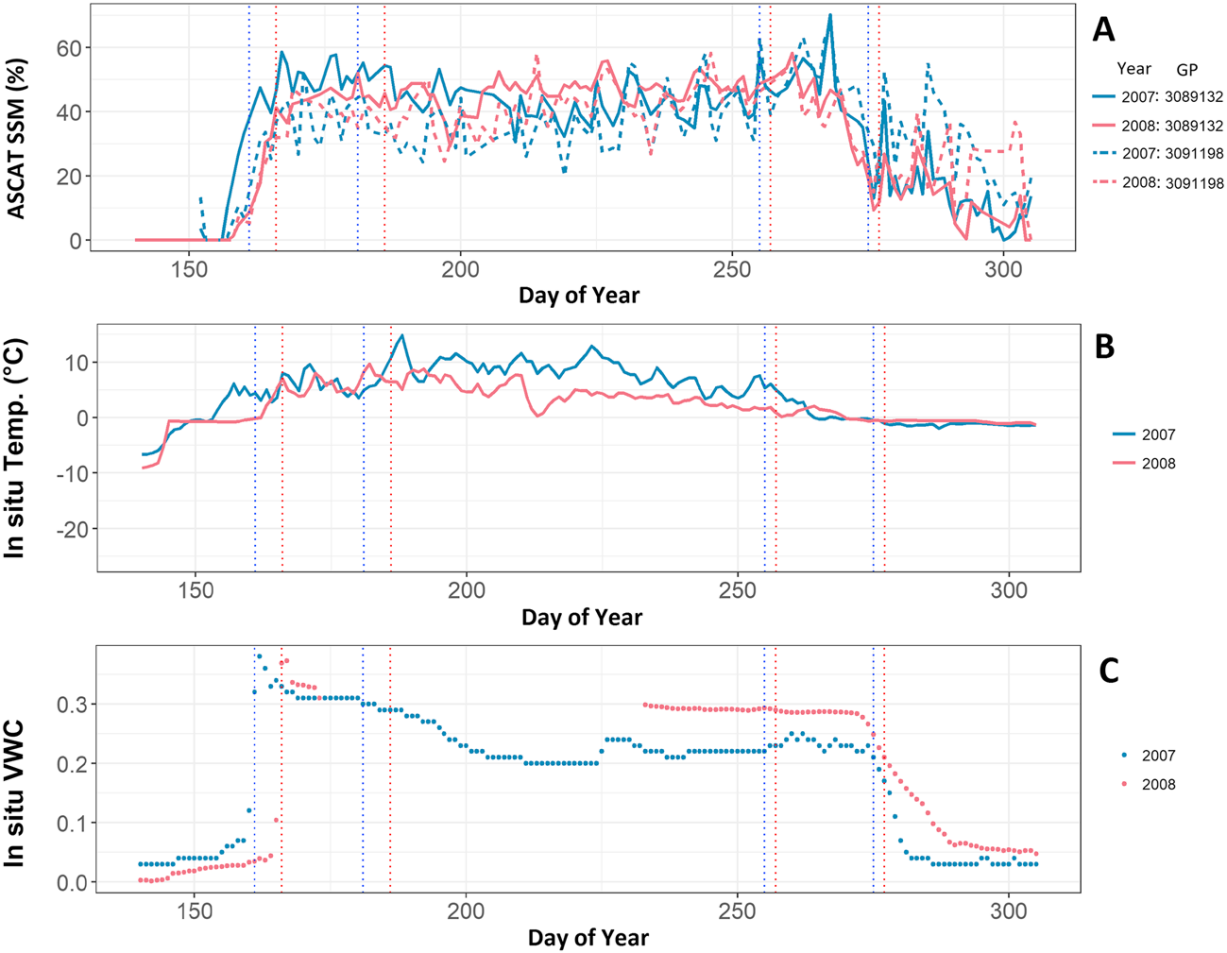
Time series of (a) ASCAT surface soil moisture (SSM), (b) in situ soil temperature, and (c) in situ VWC at Barrow during the summer of 2007 and 2008 for different grid points (3091198 over Barrow with ocean in ASCAT footprint, 3089132 south of Barrow). The sensors in panels (b) and (c) are located at 0‐cm (b) and 10‐cm (c) sensor depth. ASCAT GPI are shown according to legend. Vertical dashed lines indicate the beginning and end of time periods used for analyses in Figures 5 and 6 and Table 3. ASCAT = Advanced Scatterometer; GPI = grid cell index.

Soil temperature, which is measured at 10‐cm depth varies less at the Barrow site (Figure 4) compared to observations from the Lena Delta sites. The same applies to the soil moisture record from ASCAT measurements as well as in situ VWC. In addition, ASCAT SSM begins to decrease several days before in situ VWC values in the autumn (Figure 4).

The behavior of the soil temperature and SSM relationship over time is similar to the observations in the Lena Delta (Figure 3). There is a change to positive correlations after detrending but is less pronounced. Correlations are higher for 2008 than for 2007 (Figure 3d).

### 3.3 Quantitative Comparisons of Soil Moisture Records

Correlations between ASCAT SSM and in situ VWC vary strongly from site to site (Table 3). They can be positive or negative at the same site for different years, especially after snowmelt (average −0.15). Autumn correlations are mostly positive and also higher. The average is 0.16. Maximum values are reached at Barrow, with an average of 0.65. However, it should be noted that the VWC measurements in 2007 have gaps for the analyzed spring period. Correlations are negative for all sensors in the Lena Delta. Low (close to zero) and negative correlations coincide with high RMSE values (17–24% VWC, Table 3) derived from matched ASCAT SSM data. Barrow shows the lowest RMSE with 2.6–10.2% (VWC).

**Figure 5:**
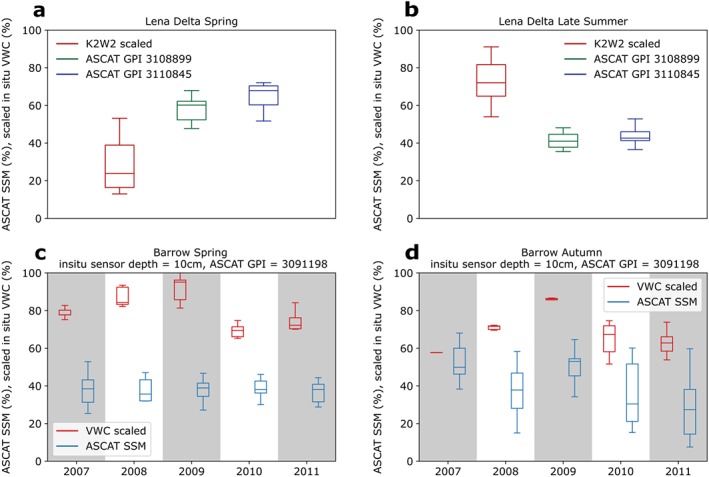
Box plots of Advanced Scatterometer (ASCAT) surface soil moisture (SSM) and in situ volumetric water content (VWC) scaled to their minimum and maximum values during the spring and autumn/late summer of 2007–2011 at the (a, b) Lena Delta and (c, d) Barrow sites. For spring, the first 20 days after thawing (determined from the in situ data) are used. For the autumn values at Barrow, the data during the last 20 days before freeze‐up (determined from the in situ data) are used. For the late summer values at Lena Delta, the data during the last 20 days of in situ data availability are used. GPI = grid cell index.

**Figure 6:**
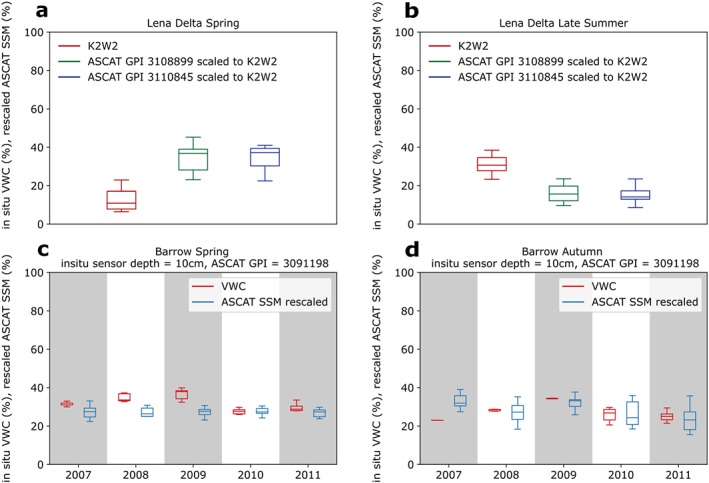
Box plots of in situ volumetric water content (VWC) and Advanced Scatterometer (ASCAT) surface soil moisture (SSM) rescaled to in situ VWC observations using mean standard deviation matching. Values are for the spring and autumn/late summer of 2007–2011 at the (a, b) Lena Delta and (c, d) Barrow sites. For spring, the first 20 days after thaw (determined from the in situ data) are used. For the autumn values at Barrow, the data during the last 20 days before freeze‐up (determined from the in situ data) are used. For the late summer values at Lena Delta, the data during the last 20 days of in situ data availability are used. GPI = grid cell index.

Boxplots for Barrow and the Lena Delta comparing the ASCAT and in situ VWC records during the first 20 days after snow clearance and soil thawing in spring, as well as the last 20 days before freezing in autumn at Barrow and last 20 days before the end of the records in the Lena Delta are shown in Figures 5 and 6. The first and last dates are indicated in Figures 2 and 4.

**Table 3:** Spring (Spr) and Autumn/Late Summer (Aut/LS) Root‐Mean‐Square Errors (RMSEs) and Pearson Correlation Coefficients of ASCAT SSM and in Situ VWC for All Sites and All Years

Site name^a^	Year	Spr RMSE	Spr Pearson R	Aut/LS RMSE	Aut/LS Pearson R
Barrow	2007	5.9	‐0.42	10.2	0.15
Barrow	2008	8.4	‐0.62	3.5	0.64
Barrow	2009	9.8	0.19	2.6	0.76
Barrow	2010	4.4	‐0.46	3.9	0.86
Barrow	2011	5.9	‐0.50	5.0	0.83
Toolik	2007	12.0	0.03	8.4	0.31
Toolik	2008	9.6	0.21	‐	‐
Toolik	2009	6.9	0.19	‐	‐
Toolik	2010	7.7	0.12	12.3	0.64
Toolik	2011	9.9	‐0.48	5.6	0.29
Sagwon	2007	34.3	0.29	19.0	0.75
Sagwon	2008	23.3	‐0.20	15.1	0.04
Sagwon	2009	23.5	‐0.02	18.6	0.15
Sagwon	2010	23.2	‐0.23	22.7	‐0.55
Sagwon	2011	21.4	0.22	8.6	0.05
Tiksi	2011	13.4	0.02	22.5	‐0.78
Tiksi	2012	11.4	‐0.10	14.0	0.32
Tiksi	2013	16.9	‐0.33	13.3	‐0.18
Tiksi	2014	5.8	‐0.29	15.5	‐0.86
LD 3108899 ‐ K2W2	2014	24.2	‐0.64	16.9	‐0.58
LD 3110845 ‐ K2W2	2014	24.0	‐0.56	17.5	‐0.44

a For Toolik in autumn 2008 and 2009, no ASCAT data were available. For the Lena Delta (LD), correlations and RMSEs were calculated between K2W2 and ASCAT GPIs 3108899, 3110845.

Our approach for scaling in situ VWC values with ASCAT SSM shows poor agreement at the Barrow site during the spring season, with approximately 20–40% difference in values (Figure 5) ASCAT SSM records are mostly below the scaled in situ values in spring but at a similar (slightly higher) level in autumn. The opposite pattern can be observed for the Lena Delta (Figure 5). However, it should be noted that values for the second period represent late summer at the Lena Delta.

The matching of ASCAT SSM to in situ VWC results in similar levels in the case of Barrow. In contrast to the VWC scaling (Figure 5), matching ASCAT SSM with in situ VWC values produces good agreement at Barrow (Figure 6). Deviations remain for Lena Delta, but reasonable agreement is obtained for the autumn period at station K3.

## 4 Discussion

### 4.1 In Situ Measurement Setup and Scales, Lena River Delta

For the ASCAT‐specific investigation in the Lena River Delta we installed VWC sensors very close to the surface within the organic layer. Tundra permafrost landscapes are fully covered by moss that overlays the mineral soil of the active layer. The organic layer in the Lena River Delta generally contains a thick moss layer (up to 30 cm) with a persistently wet 2‐ to 3‐cm‐thick fibric layer at its base. Under the moss and fibric layer a thick more decomposed organic peat layer is often present. Therefore, the installed VWC sensors do not measure *soil moisture* of a mineral soil body. Installation of VWC sensors in the mineral soil below the 20‐ to 30‐cm organic layer where the fibric layer is persistently wet would not reflect what the ASCAT senses. However, we did not find strong agreement between the time series of the in situ VWC and satellite‐derived soil moisture variations in the time period that was studied for the comparison with direct values (Table 3) nor for the median comparisons (Figure 6 and  5). The discrepancy of the temporal behavior between ASCAT SSM and in situ VWC in autumn demonstrates the limitation of the satellite information to the upper few centimeters. The soil temperature records in the soil indicate that freezing started at the surface before the decrease in VWC at 10‐cm depth. The soil temperatures remain at 0 °Celsius for several days in this case.

The Lena Delta tundra landscape is characterized by a relatively high surface water fraction which can cause a bias to the ASCAT SSM (Wagner et al., 2013). This results from variations in water surface roughness which also contribute to the backscatter signal. Such water surface roughness can be caused by wind and rain but also by variations in river discharge in the Lena Delta site (Högström et al., 2014). Figure 1b shows the amount of water bodies in each ASCAT GPI. The GPI 3110849 has a high percentage of water bodies (77%) compared to 3108899 (39%), 3110845 (24%), and 3110841 (14%) (Högström & Bartsch, 2017). GPI 3110841 would be the better choice for comparison with ASCAT SSM, but logistic limitations during fieldwork lead to the choice of the more accessible GPI 31118899.

The comparisons of the ASCAT temporal SSM dynamics to the temporal dynamics of the in situ organic layer soil temperature in the Lena Delta suggest that the increase in soil temperature and at the same time increase in backscatter and SSM, respectively, may reflect subtle changes in liquid water content through thawing and refreezing of the active layer during the first 2 weeks after the end of snowmelt. However, this is not supported by the in situ VWC data. The drying up toward autumn suggested by the ASCAT measurements could not be observed in the in situ VWC records. There is more variability for the sensors in the moss layer in direct exchange with the atmosphere than in the persistently water‐saturated fibric layer.

The correlations between the sites are relatively high, suggesting a certain level of representativeness. It has been shown that wind influences the backscatter when open water is present in the ASCAT footprint as demonstrated by Högström and Bartsch (2017) for the Lena Delta site. But this has only short‐term impacts. Comparisons of satellite‐derived soil moisture data with climate model results at high latitudes have also shown that models do not produce this *drying up* suggested by the ASCAT SSM product (Guimberteau et al., 2018). It should be noted that water logging due to local impacts of the sensor installations may also increase the saturation of the site. Water logging has been observed for sensor K1 toward the end of the summer season. Conclusions can therefore not be drawn from the data in the Lena Delta for autumn variations.

The overlying moss layer itself may also play a role with regard to the response to soil temperature in spring and changes toward autumn. The C band signal is expected to penetrate through tundra vegetation and the moss layer when it is dry. The high in situ moisture values at the deeper sensor installed in the fibric layer generally indicate very wet conditions, which is expected, as the fibric layer acts acts as the water storage layer of the moss. The backscatter range which underlies the variations in SSM in the Lena Delta is only on the order of 1–2 dB across the entire snow‐free season (Högström & Bartsch, 2017). In addition, there is low variation in in situ VWC (see Figure 2). The contribution of moisture change may, therefore, play only a minor role. The impact of soil temperature on the dielectric constant (above 0 °C) may have an effect in the case of these highly water‐saturated soils and surfaces (Mironov & Savin, 2016).

The impact of soil temperature on ASCAT SSM variations in spring is partially confirmed at the Barrow site. Barrow VWC values are similar to the Lena Delta, but ASCAT SSM levels differ. This may result from the proximity of the site to the ocean and its contribution to the backscatter. The overall low variability of soil moisture observed in the in situ records is, however, captured with ASCAT which leads to the high correlations and good results for the matching with subsequent low RMSE (3–10% for predicted VWC, Table 3). The average of the RMSE for all sites (11% in case of spring as well as autumn) does, however, not meet the general accuracy requirement for satellite‐derived soil moisture data sets, which is usually 4% (e.g., Chen et al., 2018; Sanchez et al., 2012).

### 4.2 Comparison Across Sites

The deviations which can be observed across the sites and seasons (Table 3) need to be discussed in the context of variations within the ASCAT footprint and the assumption that the chosen time period represents unfrozen conditions.

Tiksi is located close to the sea and the measurement site is in a valley surrounded by hills up to 200‐m high with bare rock surfaces. The soils at the measurement site (wetland) differ from the surrounding mountain landscape, which may lead to the low correlation and high RMSE (6–22% for predicted VWC, Table 3). Sagwon is also located in a hilly area. Bergstedt and Bartsch (2017) demonstrated a high variability of freeze/thaw transition within the ASCAT footprint at this site. This may have an impact on the accuracy of the determination of the unfrozen period as the in situ location is not representative for the ASCAT footprint.

In general, derived values from satellite measurements are not of the same order as in situ measurement (scaled between minimum and maximum). In situ values range mostly between 45% and 100% compared to ASCAT values, which range between ∼20% and 100% for the same time periods. This may result from the definition of the minimum and maximum for the scaling. This difference is similar at several sites. Only a few years have been available to define the scaling parameters for the in situ data. ASCAT dry and wet references are derived from long‐term records which also include its predecessor ERS (Naeimi, Scipal, et al., 2009), which may impact the comparability. Furthermore, the fraction of open water can impact the scaling parameters as exemplified by Högström and Bartsch (2017) for the central Lena Delta. A previous study which compared land surface model derived near SSM with ASCAT SSM also obtained a wide range of correlations, from negative to positive values, across Siberia (Gouttevin et al., 2013). The areas with negative correlations included the Lena Delta. The low correspondence in this region has been attributed to the low density of measurement sites that, in turn, drive model results. Högström et al. (2014) investigated the occurrence of these negative correlations with respect to the water fraction within the ASCAT footprint. High negative correlations coincided with areas with a high water fraction. The subsequent study over the Lena Delta (same ASCAT footprints as this study) by Högström and Bartsch (2017) showed that the high water fraction indeed causes a bias which results from wind action (causing waves which increase the backscatter). However, this bias cannot explain the negative correlations. Our results suggest that it may at least partially result from variations in the ASCAT records which are related to soil temperature variations.

Matching the ASCAT SSM to in situ data allows one to derive error measures. However, the comparably good results for Barrow need to be interpreted with care, since this site has low variability over the summer season. The high RMSE of the near‐surface VWC predicted from ASCAT soil moisture data at the Sagwon, Tiksi, and Toolik sites suggest that the error is generally much larger in tundra environments (Table 3). The higher correlations for the autumn period compared to spring indicate better applicability of ASCAT SSM information toward the end of the unfrozen period. The soil temperature influence seems to be lower. A good agreement between scaled in situ data and ASCAT SSM can also be obtained for autumn at Barrow. The record of in situ data is longer at this site which may give more reliable scaling results. However, general conclusions could not be drawn due to the low number of sites studied. More monitoring sites which provide information on multiple parameters, multiple years, and with freely accessible data are required.

K2 has the lowest SSM values of the Kurungnakh Island sites in the Lena Delta in spring. In situ soil temperatures are still close to 0 °C (for 8‐cm depth as selected for comparability to other sites, K2T2 in Figure 2) at this site which is lower than at other neighboring sites. It can be expected that the near surface was not yet fully unfrozen. This supports the findings on temporal variability, and the soil temperature dependence of the C band observations, in spring (Figure 2).

The low variation of in situ values observed at several sites and the differences between regions underline the need to use several different sites for assessments in the Arctic. The use of our own sensor setup in the Lena Delta alone would have limited the analyses to a small range of relative moisture as observed by the satellite records.

ASCAT data are only available in VV polarization which does not allow further analysis of scattering mechanisms. This needs to be further investigated with SAR. SAR also allows for better accounting of landscape heterogeneity due to the higher spatial resolution of such data (Högström et al., 2014; Wagner et al., 2008).

Results underline what has been pointed out previously by Wagner et al. (2013) regarding the necessity to build up expert knowledge in order to select those soil moisture values which are fit for application because the quality of the ASCAT SSM product varies in space and time. Disturbance of the ground through sensor installation has been identified as a critical issue for validation of near‐surface VWC values in permafrost areas.

## 5 Conclusions

Satellite SSM measurements from MetOp ASCAT, which represent relative values, can represent general differences in the degree of soil saturation between sites. We compared five sites across the Arctic in Alaska (Barrow, Sagwon, and Toolik) and Siberia (Tiksi, Lena Delta) for soil moisture and two of the sites for the relationship between ASCAT SSM and in situ temperature (Lena Delta and Barrow).

We observe low variability in ASCAT‐derived SSM and also low variability in in situ VWC of the uppermost organic layer in this type of landscape over the snow‐free season. Scaling for ASCAT SSM uses longer time series, whereas the low variability of the shorter in situ VWC time records limits the comparability of the scaling to minimum/maximum. Matching allows determination of the RMSE for the ASCAT‐derived near‐surface soil moisture in units of VWC instead of saturation. High error values suggest that ASCAT SSM values need to be treated with care across tundra environments. However, the investigated sites are all located in heterogeneous environments with a high water fraction or altitude variation in the ASCAT footprint. Continuous measurements, with multiple parameters and comparably homogeneous sites, are required for better understanding the influence of environmental factors on remotely sensed values. Soil temperature sensors distributed over a larger area could be advantageous for determining the complete thaw within the scatterometer footprint. The sensor setup in the Lena Delta, however, demonstrated the represetativeness of single‐point measurements for characterizing the temporal variability of VWC in lowland environments. The RMSE of the volumetric water content predicted by ASCAT is on average 11% across the five sites in Alaska and Siberia.

In addition to previous findings regarding the influence of near‐surface wind impacting the roughness of water surfaces, variations of C band backscatter are also impacted by soil temperature. The moss layer temperature variations are reflected in soil moisture variations derived from ASCAT during unfrozen conditions. The low variation in soil moisture over the snow‐free season may contribute to the dominance of soil temperature on the remotely sensed values at the Lena Delta as well as the Barrow site. In fact, the retrieval of soil moisture is functionally based on the relationship between soil water content and the dielectric constant. However, it also changes with soil temperature under unfrozen conditions.

## Erratum

The originally published version of this article included several minor typographical errors introduced in typesetting. These have been corrected, and this may be considered the authoritative version of record.
